# Peripheral Nerve Fibers and Their Neurotransmitters in Osteoarthritis Pathology

**DOI:** 10.3390/ijms18050931

**Published:** 2017-04-28

**Authors:** Susanne Grässel, Dominique Muschter

**Affiliations:** Department of Orthopedic Surgery, Exp. Orthopedics, ZMB/Biopark 1, University of Regensburg, 93053 Regensburg, Germany; dominique.muschter@ukr.de

**Keywords:** osteoarthritis, neurotransmitters, peripheral nervous system, cartilage, subchondral bone, CGRP, substance P, adrenoceptors

## Abstract

The importance of the nociceptive nervous system for maintaining tissue homeostasis has been known for some time, and it has also been suggested that organogenesis and tissue repair are under neuronal control. Changes in peripheral joint innervation are supposed to be partly responsible for degenerative alterations in joint tissues which contribute to development of osteoarthritis. Various resident cell types of the musculoskeletal system express receptors for sensory and sympathetic neurotransmitters, allowing response to peripheral neuronal stimuli. Among them are mesenchymal stem cells, synovial fibroblasts, bone cells and chondrocytes of different origin, which express distinct subtypes of adrenoceptors (AR), receptors for vasoactive intestinal peptide (VIP), substance P (SP) and calcitonin gene-related peptide (CGRP). Some of these cell types synthesize and secrete neuropeptides such as SP, and they are positive for tyrosine-hydroxylase (TH), the rate limiting enzyme for biosynthesis of catecholamines. Sensory and sympathetic neurotransmitters are involved in the pathology of inflammatory diseases such as rheumatoid arthritis (RA) which manifests mainly in the joints. In addition, they seem to play a role in pathogenesis of priori degenerative joint disorders such as osteoarthritis (OA). Altogether it is evident that sensory and sympathetic neurotransmitters have crucial trophic effects which are critical for joint tissue and bone homeostasis. They modulate articular cartilage, subchondral bone and synovial tissue properties in physiological and pathophysiological conditions, in addition to their classical neurological features.

## 1. Introduction

Osteoarthritis (OA) is an age-related and/or trauma-induced multi-factorial, slowly progressing and primarily non-inflammatory degenerative disorder of the synovial joints, culminating in the irreversible destruction of the articular cartilage. Although OA is the most common musculoskeletal condition that causes significant health and social problems worldwide, its exact etiology is still unclear. Age-related wear, overuse, limb mal-alignment and genetic disorders, as well as metabolic problems (obesity, immune responses, diabetes), play important roles in the onset of OA [1,2]. OA is not exclusively a disorder of articular cartilage; it can be considered as an organ failure of the whole joint with additional abnormalities especially in bone, ligaments, synovium and the joint capsule [3]. Clinical symptoms of OA appear in more than 10% of the world population and affect almost everyone over the age of 65. As a consequence of the increasing longevity and obesity within the European Community, the economic and social burden caused by OA is growing rapidly and substantially influencing the life quality of affected individuals, with enormous costs to the health care system with respect to diagnosis, treatment, sick leave, rehabilitation, and early retirement [4]. OA of the hips and knees tends to cause the greatest burden to the population as pain and stiffness in these large weight-bearing joints often lead to significant disabilities requiring surgical intervention [5]. A recent survey in 15 European countries revealed that on average 19% of the population suffers from chronic pain, most frequently caused by disorders of the musculoskeletal system, specifically OA, herniated and/or deteriorating discs, traumatic injury and rheumatoid arthritis [6].

Sympathetic and sensory nerve fibers innervate synovium, trabecular and subchondral bone, bone marrow, periosteum and fracture callus [7,8]. Vascularization of the growth plate and matrix differentiation during endochondral ossification in embryonic limb development are dependent on the peripheral nervous system [9] which suggests a critical role in skeletal growth and limb formation processes. Recently, our group demonstrated that femoral bone of substance P-deficient and sympathectomized mice has inferior mechanical strength, reduced bone mass and trabecular numbers [10].

Unlike other musculoskeletal connective tissues such as bone, periosteum and synovium, healthy cartilage does not contain blood vessels and is not innervated by nerve fibers, indicating that cartilage for some reason might be a hostile environment for spreading of nerve fibers. However, despite lack of nervous innervation, cartilage metabolism is modulated and influenced by neurotransmitters released either from nerve fibers located in neighboring tissue or directly from chondrocytes (for review see [11,12]).

This review focusses on recent literature describing effects of sensory and sympathetic nerve fibers and their neurotransmitters on joint tissue pathophysiology in OA affecting cartilage and bone turnover.

## 2. Sensory and Sympathetic Nerve Fibers in Joint Physiology

Sometimes, sensory nerve fibers are observed in contact with a subpopulation of chondrocytes located in growth cartilage and at the surface of articular cartilage [13,14]. Calcitonin gene-related peptide (CGRP)-positive fibers, which originate from the periosteum and near insertion regions of muscle and tendons, innervate up to 25 μm into the articular and meniscal cartilage tissues in rat knee joints. These fibers are observed between individual chondrocytes, suggesting a local trophic function different from their classical roles. However, subpopulations of substance P (SP)-positive axons in perichondrium and periosteum have been detected which do not innervate the cartilage. So far structural or molecular differences to those fibers which innervate cartilage have not yet been described. CGRP- and SP-positive nerve fiber innervation precedes the development of cartilage canals shortly after birth [15,16]. These fibers were detected inside the canals of growth cartilage in the epiphysis of young rats, thus allowing physical contact to chondrocytes. Cartilage canals which are invaded by sensory nerve fibers precede the development of the secondary ossification center. Possibly, these nerve fibers modulate the formation of synovial joints by releasing trophic factors [17]. The authors observed that the inner layer of the perichondrium is richly innervated by peripheral nervous fibers. These fibers were often found at the interface with the cartilage, in close contact with the outer layers of epiphyseal chondrocytes which actively produce cartilaginous matrix and are arranged in clonal grouping. These observations imply important functions of sensory nerve fibers for regulating chondrogenic differentiation during limb growth in embryonic development.

Furthermore, bone and joint tissues like the synovium are densely innervated by sympathetic nerve fibers. Tyrosine hydroxylase (TH)-positive catecholaminergic nerve fibers are mainly associated with blood vessels but periosteal and bone-adjacent ligamental structures also contain free nerve endings [18,19]. Nerve fibers showing immunoreactivity for the sympathetic neuropeptide vasoactive intestinal peptide (VIP) were detected near epiphyseal trabecular bone, the periosteum and also in the bone marrow compartment [20,21]. Work from Serre et al. identified an extensive network of neuronal cell processes positive for the neuronal marker neurofilament 200 in the long bones of neonatal rats which revealed tyrosine-hydroxylase (TH)- and VIP-immunoreactivity [22]. These nerve processes coming in close contact with bone cells and medullary cells indicate a regulatory role for sympathetic nerve fibers on bone cell activity during development. The importance of sympathetic innervation for bone homeostasis is undisputed but remains controversially discussed because stimulation and inhibition of the sympathetic nervous system elicits anabolic as well as catabolic effects, being strictly context-dependent [23,24,25].

## 3. Sensory and Sympathetic Nerve Fibers in Osteoarthritic Joints

### 3.1. Sensory Nerve Fibers and Neuropeptides

Suri et al. have localized both sensory (SP- and CGRP-positive) and sympathetic nerve fibers (neuropeptide Y (NPY)-positive) in the articular cartilage in human tibiofemoral OA [26]. In addition, they have detected blood vessels and nerves at the osteochondral junction and in osteophytes at the human tibiofemoral joint. These nerve fibers were present within vascular channels in both mild and severe OA-stages. It seems that in the pathogenesis of OA, fine unmyelinated nerves grow into joint structures through vascular channels, mainly from subchondral bone breaching through the tidemark rather than coming from synovium or periosteum.

The exclusively perivascular localization of nerves in the surface layer of articular cartilage implies that vascularization is the driving force behind its innervation and without angiogenesis no nerve fibers can grow into cartilage [26]. Free nerves, not associated with blood vessels, were not observed within the articular cartilage. Vascularization of the non-calcified cartilage was found throughout a wide range of histological OA stages and was not restricted to end-stage OA. Possibly, scoring of nervous innervation and degree of vascularization of cartilage might be exploited as a measure for severity of degradative changes in OA pathogenesis or even staging of disease progression. However, the mechanisms that drive innervation of cartilage in OA have remained incompletely understood up to now.

It is reported that in patients with knee OA, vascularization and accompanying innervation of the articular cartilage might be a source of pain. Both sympathetic nerves and perivascular sensory nerves innervate tibial osteophytes, with the sensory nerves detected at the base of osteophytes [26,27]. Radiological grading of osteophytosis is associated with reported pain severity in OA. The reason may be the observed sensory innervation of osteophytes. Chronic pain sensation in OA is of profound clinical importance but pain mechanisms are poorly understood. To date it has not been clarified which joint tissues give rise to OA pain, and the nature of OA pain (nociceptive versus neuropathic) is still debated [28]. One tissue compartment, which can be a source of pain in OA, is the osteochondral junction. Interestingly, angiogenesis seems to predominate in both early and late OA, although, besides proangiogenic factors, antiangiogenic factors are also upregulated in both OA stages as observed in rats [29,30]. In OA, proangiogenic factors (such as vascular endothelial growth factor, VEGF) are produced by chondrocytes [31] and the synovium [32,33] as well as in the subchondral bone, marrow spaces and adjoining osteochondral channels. Chondrocytes in healthy joints produce antiangiogenic factors, such as troponin I [34] and thrombospondin [35]. In OA, new blood vessels that cross the tidemark in articular cartilage might be associated with sensory nerves, as might those in developing osteophytes [26] and those that penetrate the inner regions of the meniscus [36]. Pain, the most common problem of patients with arthritis (both rheumatoid arthritis (RA) and OA), is mediated by sensory nerves. Perivascular nerve growth might contribute to pain in OA because nerve growth is associated with peripheral sensitization, and nerves in structures such as cartilage that are not normally innervated could be exposed to chemical stimulation and mechanical stress. Thus, neovascularization may contribute to pain in patients with OA because of the accompanying sensory innervation. In addition, innervation of other joint structures may contribute to OA pain. Ikeuchi et al. reported that in spite of a significant decrease in total innervation in OA knees, the posterior cruciate ligaments (PCLs) have constant nociceptive sensory innervation. In PCLs, constantly CGRP-immunoreactive nerve fibers in both OA and non-OA knees, were detected, even though the PCLs in OA knees were statistically less innervated than non-OA knees. Although the relationship between the decrease in total innervation in PCLs and OA pathophysiology is still unclear, the PCL qualifies as the possible source of OA knee pain [37]. Clinical data from OA patients support an association between CGRP-immunoreactive fibers in the joint and pain. Ashraf et al. demonstrated CGRP-positive sensory nerve profiles in the outer region of menisci, and most of these nerves were associated with blood vessels [36]. Perivascular CGRP-positive nerve fibers are increased in density in the menisci and joint capsules of patients with high chondropathy scores and thus severe OA pathology. CGRP is co-localized with SP in unmyelinated sensory nerve fibers, and therefore extends previous findings that perivascular nerves containing SP were located in the peripheral portion of torn menisci. A study from Saxler et al. provides the first immunohistochemical evidence that the innervation density of SP- and CGRP-immunoreactive nerve fibers in the human hip joint is increased in patients with painful osteoarthritis. Patients with painless hip failure completely lacked SP- and CGRP-positive fibers surrounding the joint [38,39].

During pathogenesis of OA, osteochondral angiogenesis is associated with increased nerve growth factor (NGF) expression in subchondral spaces, vascular channels and in chondrocytes themselves [32]. Besides NGF synthesis, production of vascular endothelial growth factor (VEGF) and platelet-derived growth factor (PDGF) were also associated with chondrocytes. VEGF-positive chondrocytes were predominantly localized to the superficial zone of articular cartilage of OA samples and NGF-positive chondrocytes were localized to superficial but not deep articular cartilage zones in OA. This NGF immunoreactivity was co-localized with CGRP-immunoreactive nerve fibers in the same vascular channels. Increased NGF production may thus contribute to OA pain, both structurally (increased aberrant innervation at the osteochondral junction) and through peripheral sensitization. This was confirmed in studies in human OA material, showing that increased vascular penetration and density of perivascular CGRP—positive sensory nerve profiles in the meniscus are a potential source of pain in knee OA and lead in addition to propagation of further inflammation and tissue damage [36,40].

**Figure 1:**
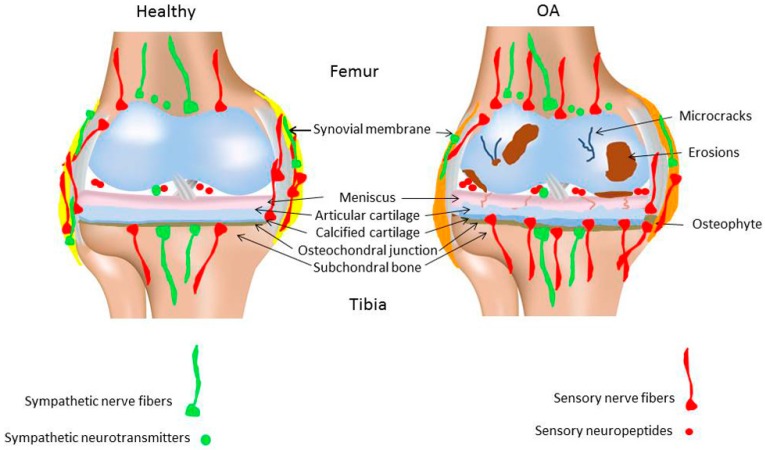
Proposed alteration of sensory and sympathetic nervous joint innervation in osteoarthritis. It is discussed that there is an increase of sensory nerve fiber innervation in the subchondral bone zone which reaches into the calcified cartilage zone from the subchondral bone or which comes into close contact to the articular cartilage. On the contrary, synovial sensory innervation decreases in an early osteoarthritis (OA) stage and presumably also in later OA stages. In addition, concentration of sensory neuropeptides in synovial fluid increases with increasing OA severity. In contrast, sympathetic innervation is presumably not profoundly changed during OA pathology. Modified from [12].

It is unclear if sensory nerve fiber density during OA pathogenesis changes and whether the sensory fiber profile is different from healthy joints. In a murine OA model (intraarticular injection of collagenase), CGRP- and SP-positive nerve fibers disappeared 5 weeks after induction of OA [41] In a similar OA model, Murakami et al. reported that the densities of protein gene product (PGP) 9.5- and CGRP-positive nerve fibers in the synovium were drastically decreased just one week after the collagenase injection [42]. This aggressive and painful method of OA induction leads to various degenerative alterations in synovial joint tissues (among other soft tissues) after a few weeks. In contrast to these reports, some studies on human knee OA revealed an increase in SP- and CGRP-positive nerve fiber density in the synovial tissue [38,39,43], whereas others have described a decrease of these nerve fibers in the synovium [44]. As most of these studies are undertaken in late-stage chronic OA, there is not much known about a potential association between the pathological changes in knee tissues and changes in these nociceptive fibers innervation profile during the early acute phase of the disease. It is likely that the normal vascular and neural network in early or late OA stages is altered, however, the underlying molecular mechanisms have not yet been identified. In the same line, it is unknown if nerves are destroyed as a consequence of OA or if pathogenesis of OA is facilitated due to nerve disappearance (Figure 1).

It was reported earlier that in a spontaneous age-related OA mouse model, loss of SP- and CGRP-positive innervation from the whole joint always preceded cartilage degeneration [45]. Immunocytochemistry showed that the percentage of neurons expressing SP and CGRP increased with age, whereas the spontaneous loss of general joint innervation and of the relative increase in peptide-expressing neurons occurred in the first year of life. Histological examination of knee joints of mice at various ages showed that loss of joint innervation always preceded histological changes of cartilage degeneration and surgical denervation accelerated cartilage degradation. The mice usually developed a mild form of osteoarthritis, but surgical ablation of joint innervation caused the development of severe patellofemoral OA. These findings would be consistent with the hypothesis that an age-related loss of sensory joint innervation may contribute to the development of OA. Whether sensory nerve fibers are lost, remain unaltered, increase or change tissue distribution as a prerequisite of OA pathogenesis in humans remains to be determined. Notably, CGRP and SP synovial fluid levels are increased in developmental dysplasia of the hip. CGRP concentration in synovial fluid is increased in knee OA patients and clearly correlated with increased Kellgren–Lawrence (KL) scores (indicating OA severity) [46,47,48]. Further insight into these mechanisms and relations requires reproducible OA-animal models resembling slow proceeding pathogenesis of human OA and enabling longitudinal studies from early onset of the disease to late stages.

### 3.2. Sympathetic Nerve Fibers and Neurotransmitters

Compared to sensory innervation, the influence of OA progression on sympathetic nerve fiber distribution and signaling or vice versa, is less well known and deserve more intense examination.

In classical inflammation-driven diseases like RA, the local synovial innervation pattern is subjected to massive remodeling during disease progression. Synovial specimens from RA and OA patients undergoing knee joint replacement were compared for the expression of TH-positive and SP-positive nerve fibers. In RA synovium a significantly lower number of TH-positive nerve fibers and a significantly higher number of SP-positive nerve fibers were detected compared to OA patients. Whereas the number of TH- and SP-positive nerve fibers was similar in OA patients, RA patients showed a marked preponderance of sensory over sympathetic nerve fibers [49]. Both diseases are associated with enhanced pain perception, but in OA-affected synovium the increase of sensory innervation is quite reduced compared to RA. One possibility as to why OA progression is not accompanied by pronounced changes in synovial innervation pattern might be that in RA, nerves play a decisive role in perpetuation of the inflammatory response, whereas in OA inflammation does play an important but less pointed role and the innervation profile might undergo more subtle changes (Figure 1).

In an experimental rat model of temporomandibular joint OA, rats showed a robust sprouting of TH-positive nerve fibers and increased norepinephrine (NE) levels in the condylar subchondral bone compartment compared to control rats. This is in line with accelerated subchondral bone loss, but so far no such observations have been reported for other OA joints [50]. The subchondral bone of rat knee joints with monoiodacetate-induced OA showed clear sprouting of CGRP- and tropomyosin receptor kinase A (TrkA)-immunoreactive fibers and this phenomenon was associated with increased pain [51] (Figure 1). Interestingly, Ghilardi et al. demonstrated that in a mouse model of chronic arthritic pain induced by Complete Freund Adjuvant (CFA) injection into the knee joint, the sprouting of CGRP-positive sensory fibers in the synovium is accompanied by sprouting of TH-positive sympathetic fibers in close proximity [52]. A more elaborated analysis of sympathetic nerve fiber profile in different joint compartments in OA might therefore help understanding pain behavior, but might also contribute to our knowledge about metabolic processes in OA with regard to sympathetic neurotransmitter activity.

Due to the sparse availability of healthy human tissue for comparative analysis in pathophysiological processes, Eitner et al. analyzed nerve fiber distribution in normal joints from rats and sheep and compared their findings to human OA tissue samples [53]. They identified a dense capillary network accompanied by a sympathetic and sensory neuronal network in normal synovial tissue of rat and sheep joints. Non-inflamed tissue samples of OA patients showed a similar pattern but according to the grade of inflammation of other tissue regions of these patients, the vascular and neuronal network decreased and disappeared. Various degrees of local inflammation might therefore evoke changes in innervation pattern of OA synovium comparable to RA and might also induce TH expression in joint cells, providing a local source for NE as has been shown for synovial macrophages [49]. In this line, our group demonstrated that chondrocytes of OA cartilage express TH in all three cartilage zones with the weakest staining intensity in the deep zone [54]. With that, the cells possess the prerequisite to induce an anti-inflammatory response in their immediate environment, perhaps serving as a primarily protective measure in OA.

A recent review extensively elucidated the role of VIP in OA pathology [55]. The authors deduced protective VIP characteristics in OA comparable with RA from the VIP content in OA synovial fluid and articular cartilage which negatively correlated with progressive joint damage and disease severity. The review also mentions one contradictory study which implicates VIP application in the OA knee joint as a potential source of knee pain by sensitizing afferent nerves and enhancing the nerve fire rate. In this particular study, intra-articular injection of VIP caused a rapid but transient algesic effect evaluated by hind limb incapacitance measurements [56]. Furthermore, administration of the VIP receptor antagonist VIP_6–28_ into the knee joint of rats with monoiodacetate-induced arthritis was able to reduce this pain behavior, thereby showing for the first time that peripheral application of VIP causes increased knee joint allodynia and secondary hyperalgesia. The anti-inflammatory and anti-catabolic properties of VIP, which were demonstrated in arthritis before, might offer therapeutic potential in OA, but the ability of VIP to promote hyperalgesia in OA joints needs to be more carefully evaluated in order to consider VIP-targeted therapies in OA.

NPY-positive sympathetic nerve fibers were detected at the base of tibial osteophytes, the subchondral bone marrow and within vascular channels of articular cartilage in OA. They were similar in number to sensory nerve fibers in all compartments analyzed [26]. Differential NPY concentrations might represent a potential candidate as an OA biomarker due to known effects on pain perception. In a study including 100 OA patients with varying grades of Watanabe pain scores and radiographic stages, increasing synovial fluid concentrations of NPY correlated with increasing pain scores [57]. NPY concentrations in OA patients were significantly higher compared to controls and also middle and advanced stage OA patients exhibited higher NPY synovial fluid concentrations compared to early-stage OA patients. NPY effects can therefore be associated with OA pain but the synovial fluid levels do not correlate with radiographic OA stages. Additionally, to the classical pain-associated neurotransmitters in OA, NPY might further contribute to pain but the exact mechanisms need future examination.

## 4. Sensory and Sympathetic Neurotransmitters and Their Receptors in Chondrocytes

### 4.1. Sensory Neurotransmitters

**Figure 2:**
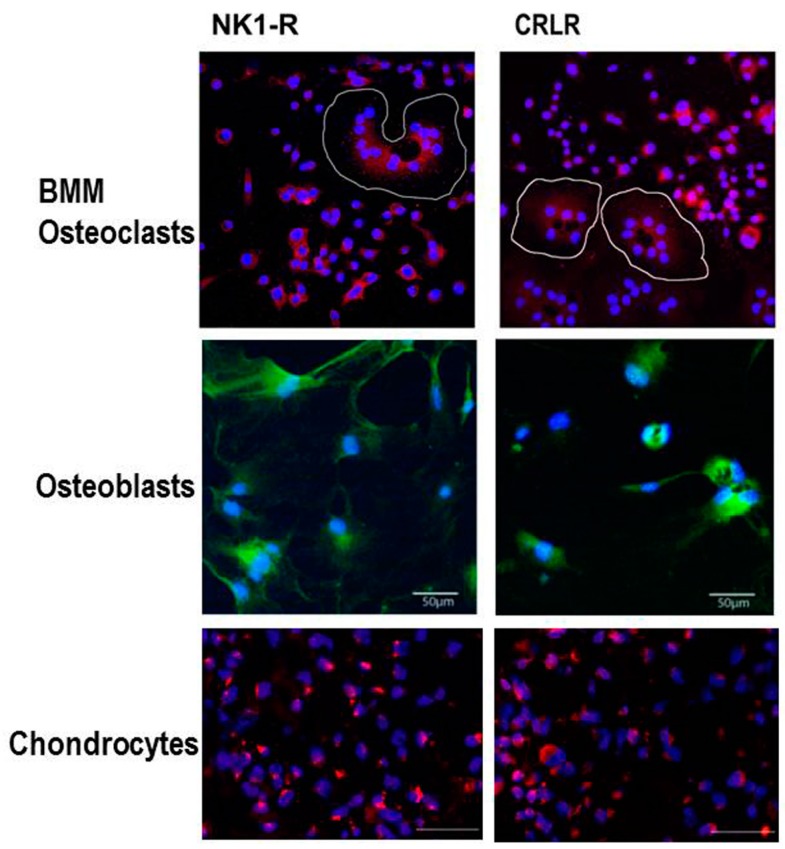
Receptors for sensory neuropeptides. On bone marrow-derived macrophages, osteoclasts (white circles), and chondrocytes from OA cartilage (red fluorescence, detection by Alexa568-coupled secondary antibody) as well as osteoblasts (green fluorescence, detection by Alexa488-coupled secondary antibody) we detected receptors for SP (neurokinin 1 receptor, NK1-R) and calcitonin gene-related peptide, CGRP (calcitonin receptor-like receptor, CRLR). Receptors for sympathetic neurotransmitters expressed in chondrocytes and bone marrow-derived macrophages (BMM)/osteoclasts were previously published [54,62]. SP = Substance P.

**Figure 3:**
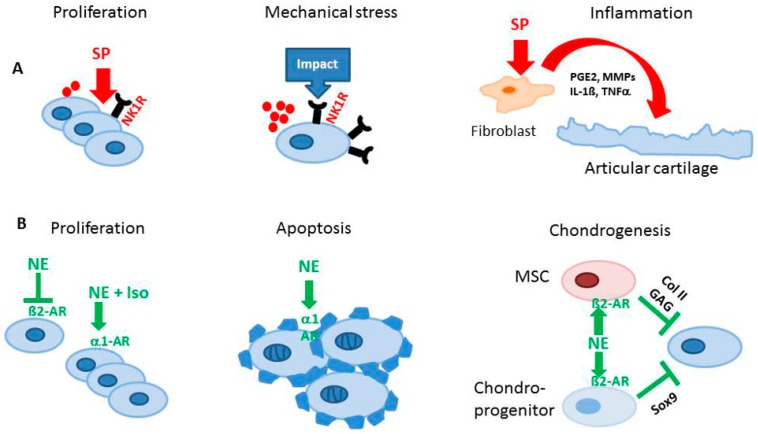
Effects of sensory and sympathetic neurotransmitters on chondrocytes. Changes in local innervation pattern of sensory and sympathetic nerves related to OA leading to altered neuropeptide microenvironments can alter chondrocyte behavior and metabolism contributing to the observed OA phenotype. (**A**) Substance P increases proliferation of chondrocytes indicating anabolic effects. Application of mechanical load increases expression of NK1-R and endogenous synthesis of substance P. Stimulation of synovial fibroblasts with SP induces release of inflammatory mediators, promoting cartilage degradation. (**B**) β2-AR signaling inhibits proliferation of chondrocytes whereas α1-AR signaling induces proliferation, implying dual effects of the sympathetic nervous system. NE signalling via α1-AR induces apoptosis of chondrocytes and signal transduction via β-AR inhibits chondrogenic differentiation of MSC and chondroprogenitor cells. GAG: Glycosaminoglycans; Col II: Collagen II; SP: Substance P; PGE2: Prostaglandine E2; MMP: Matrix metalloproteinase; IL: Interleukin; TNF: Tumor necrosis factor; NE: Norepinephrine; AR: Adrenoceptor; Iso: Isoproterenol; NK1-R: Neurokinin receptor 1; MSC: Mesenchymal stem cell.

Besides their classical function in nociception, SP and CGRP appear to have extra functions in the musculoskeletal system. Previously, we described that newborn murine costal and adult human articular chondrocytes endogenously produce SP and its neurokinin-1 receptor (NK1-R) [58]. Our data suggest that also chondrocytes isolated from human OA and non-OA articular cartilage produce SP and CGRP and carry receptors for both neuropeptides, the NK1-R and the calcitonin receptor-like receptor (CRLR) (Figure 2). Expression of SP and its receptor was increased in chondrocytes and even within the cartilaginous extracellular matrix after low-impact regimented exercise, indicating a role in signaling pathways through which chondrocytes respond to mechanical stimulation. Blockade of SP signaling by a chemical antagonist of the NK1-R inhibited chondrocyte responses to mechanical stimulation (Figure 3). This was demonstrated by Millward-Sadler et al. who suggested that SP is involved in mechanotransduction via the NK1-R [59,60]. In these experiments, SP was necessary to elicit a hyperpolarization response of the cell membrane and, concomitant changes in gene expression as response to mechanical stimulation indicate a role of SP in maintenance of articular cartilage matrix integrity and function after mechanical stress. The same group demonstrated that normal and OA chondrocytes reacted differently to mechanical stimulation in that OA chondrocytes upregulated gene expression of the SP encoding gene, tachykinin *(TAC) 1*, whereas non-OA chondrocytes did not show this phenomenon [61]. Notably, the transcription factor termed the neuron restrictive silencer factor (NRSF) and the truncated splice variant, NRSF short form (sNRSF) which both are major modulators of preprotachykinin A (*TAC1*) gene expression, were upregulated after mechanical stimulation in OA chondrocytes only. This differential expression of *TAC1* and *sNRSF* in OA chondrocytes suggests an association of their expression with the disease. The change in expression of *sNRSF* and *TAC1* mRNA following mechanical stimulation in OA but not normal chondrocytes suggests that sNRSF may be involved in the regulation of SP production in OA cartilage and might qualify as predictive marker for diseased cartilage.

In addition, we recently demonstrated that costal chondrocytes isolated from newborn mice increased their proliferative activity when stimulated with SP, which also increased cell-matrix adherence by inducing formation of focal adhesion contacts [58] (Figure 3). Our observation implies that SP might modulate proliferation rate of growth plate chondrocytes and consequently the terminal differentiation process during endochondral ossification, affecting longitudinal growth. Data from our group demonstrate expression of SP and NK1-R in the hypertrophic zone of growth plate chondrocytes [12]. It is thus conceivable that in chondrocyte physiology and in chondrogenic differentiation during skeletal growth, endogenous SP acts mainly as a trophic, anabolic factor and does not function as a classical neuropeptide.

However, in adults, the detection of higher levels of SP in synovial fluid from patients with RA and OA, and increased expression of NK1-R, indicates possibly catabolic effects of SP on articular cartilage [63]. In other musculoskeletal diseases such as developmental dysplasia of the hip, increased levels of SP and αCGRP detected in synovium and synovial fluid indicate also catabolic and pro-inflammatory effects of these neuropeptides [47]. αCGRP concentrations in human serum and synovial fluid correlate with increasing KL grade and are lowest in controls without OA diagnosis [46]. In addition, transforming growth factor (TGF)-β and basic fibroblast growth factor (bFGF) play an important role as inductor or promoter for production of SP in synovial fibroblasts. These data are supported by Im et al. who elegantly demonstrated that SP induces interleukin (IL)-1β release [64] (Figure 3). The authors propose a mechanism by which bFGF, together with SP, reduce proteoglycan deposition and stimulates production and release of matrix metalloprotease (MMP)-13 in human articular chondrocytes and thus accelerates catabolic processes in cartilage.

It is very likely that SP has autocrine functions and modulates physiological metabolism of chondrocytes and cartilage homeostasis during skeletal growth different from pathophysiology. In synovial cells, SP is a potent mediator of inflammation by promoting secretion of prostaglandin E2 (PGE2), several MMPs [65], reactive oxygen species [66], IL-1β and tumor necrosis factor (TNF)-α [67]. Similarly, SP might induce catabolic pathways in chondrocytes and promote cartilage degradation (Figure 3).

**Figure 4:**
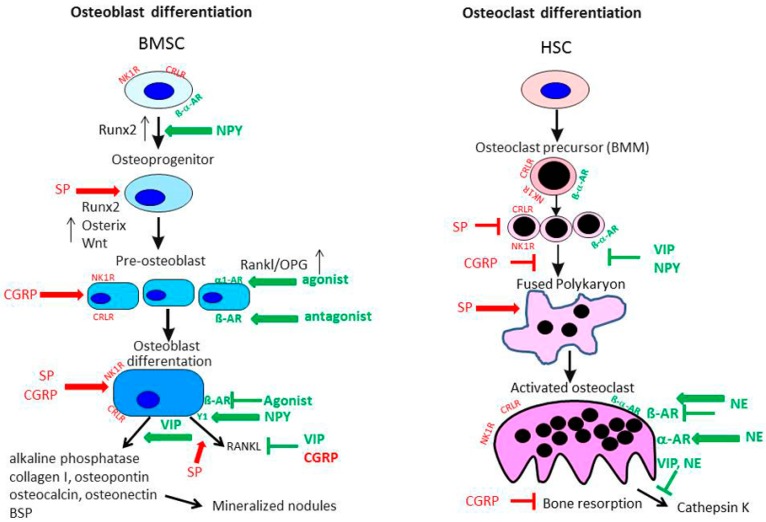
Effects of the sensory and sympathetic nervous system on osteoblast and osteoclast metabolism. Both sensory and sympathetic neurotransmitters affect the differentiation process and activity of osteoblasts and osteoclasts/BMM in many different ways. Modified from [11]. BMM: bone marrow-derived macrophages; SP: substance P; NE: norepinephrine; AR: adrenoceptor; NK1-R: neurokinin receptor 1; BMSC: mesenchymal stem cell; NPY: neuropeptide Y; CGRP: calcitonin gene-related peptide; VIP: vasoactive intestinal peptide; OPG: osteoprotegerin; Rankl: receptor activator of nuclear factor κB ligand; CRLR: calcitonin receptor-like receptor; BSP: bone sialoprotein; HSC: hematopoietic stem cells.

To date, there are no reports listed in PubMed with respect to production of CGRP and its receptor consisting of the two components CRLR/receptor activator modifying protein (RAMP) 1 in cartilage. However, we demonstrated expression of CRLR in articular cartilage chondrocytes obtained from OA and non-OA patients mainly in middle and deep zones similar to the NK1-R [12]. In bone metabolism αCGRP is described as an anabolic factor which stimulates osteoblast activity and consequently bone formation [21,68] (Figure 4). Possibly, αCGRP has similar anabolic effects in cartilage physiology. Of note, inhibition of αCGPR effects by blocking its receptor with an antagonist, attenuated subchondral bone sclerosis in a murine surgical OA model (destabilization of the medial meniscus) indicating a dual role of CGRP in pathophysiology as reported for SP [69]. Consequently, articular cartilage erosion and degeneration was delayed in the early stage in this OA model but in a later stage of the disease these effects were abolished (8 weeks after OA induction). It is likely that additional factors affect bone turnover and compensate for αCGRP effects.

### 4.2. Sympathetic Neurotransmitters

Sympathetic nerve fibers and/or neurotransmitter-producing cells are present in different joint tissues, except in articular cartilage and the avascular zone of the meniscus [49,70]. These data indicate that neurotransmitters released into the synovial fluid can influence cartilage tissue and chondrocyte metabolic activity provided that specific adrenergic neurotransmitter receptors are present on chondrocytes.

In neonatal mouse tibial sections, β2-adrenoreceptor (AR) and α2A-AR mRNA expression was detected in chondrocytes by in situ hybridization [71]. All other adrenergic receptors were not present. Similarly, our group described that β2-AR and α2A-AR mRNA but also α1B-AR and α1D-AR were expressed in newborn murine costal chondrocyte cultures [58]. An earlier study performed by Lai et al. confirmed only the expression of β2-AR on growth plate chondrocytes from ribs of embryonic E18.5 mice [72]. Studies on cartilage tissue explants isolated from OA patients after endoprothetic surgery and on chondrogenic progenitor cells obtained from human OA cartilage explants revealed that also under degenerative conditions, β2-AR and α2-AR are present [54,70]. As it is suggested that many biomechanical pathways in OA pathogenesis are altered, it is conceivable that β2-AR might contribute to disease development related to overloading of cartilage, because β2-adrenergic drugs have been shown to influence mechanical events in bone tissue [21,73]. Cumulative genetic evidence strongly supports the biological relevance of β2-AR signaling in the regulation of bone remodeling [73] (Figure 4). The observation that osteoblast-specific inactivation of the β2-AR induces a high bone mass phenotype confirmed the osteoblast-specific role of the β2-AR in the regulation of bone remodeling and supported the notion that sympathetic signals, mainly via the β2-AR expressed in osteoblasts, restrain bone formation and favor bone resorption [74].

Peptidergic NPY receptors play an important role in bone homeostasis [75,76] but no data exists about chondrocytes expressing NPY receptors. Only one study so far has confirmed the existence of the Y1 receptor on bone marrow stromal cells being able to contribute to cartilage repair processes [75]. Similarly, no studies have described the presence of VIP receptors on chondrocytes until now.

Sympathetic neurotransmitters control chondrogenic progenitor cell differentiation and mature chondrocyte function but up to now adrenoreceptor expression levels were not compared between healthy and pathological conditions. Bone marrow-derived stem cell (BMSC) migration from the bone marrow towards adjacent tissues is affected by catecholamines either released by sympathetic neurons or by immune cells. Besides sympathetic nerves located in the bone marrow, resident bone marrow cells also synthesize substantial amounts of catecholamines [77,78]. Thus, increased sympathetic neurotransmiiter concentrations during stress or in an inflammatory situation might critically influence progenitor cell physiology in the bone marrow. Moreover, NE (and dopamine) release is subject to a circadian rhythm with early morning peaks. Furthermore, treatment of murine pre-chondrogenic cells with epinephrine resulted in β2-AR-dependent inhibition of *Sox9* gene expression and consequently of chondrogenic gene expression via classical Gαs-cyclic adenosin mono phosphate (cAMP) signaling [71] (Figure 3). Similarly, NE inhibited chondrogenic differentiation of BMSC and OA-cartilage-derived progenitor cells [70]. Via β2-AR-signaling, NE in high concentrations repressed collagen II and glycosaminoglycan deposition (Figure 3) and accelerated the expression of hypertrophic markers like collagen X and MMP-13. NE in low concentrations, acting preferentially via α-AR, had no effects.

In contrast to findings in progenitor cell chondrogenesis studies, Lai et al. reported that the specific β2-AR agonist isoproterenol inhibited indian hedgehog (IHH) and collagen X expression via cAMP and extracellular signal-regulated kinases (ERK)-1/2 activation in the murine growth plate [72]. Furthermore, isoproterenol stimulated the proliferation of chondrocytes (Figure 3). In a follow-up study, these authors elaborated on the involvement of the transcription factor Jun-B, activated by β2-AR in chondrogenesis, inhibiting the expression of Sox6 and collagen II [79] similarly to mesenchymal stem cells (MSCs) [70]. In costal chondrocytes isolated from newborn mice, apoptosis decreased after NE stimulation, however, extracellular matrix formation with respect to collagen and proteoglycan production was not influenced by NE in that experimental setup [58] (Figure 3).

NE modulates the function of human OA chondrocytes. In OA, inflammation and activation of the innate immune system is recognized as an important hallmark. However, not so much is known about the effect of inflammation on chondrocyte function in OA. For instance, Lorenz and colleagues investigated the effects of NE together with an inflammatory stimulus like IL-1β, simulating an early OA microenvironment [54]. NE in high concentrations decreased chondrocyte proliferation via β2-AR signaling. In contrast, NE in low concentration increased the proliferation of OA chondrocytes via β1-AR signaling, suggesting that NE might exhibit dual effects on chondrocyte proliferation in OA depending on sympathetic activity (Figure 3). In addition, NE reversed IL-1β-mediated suppression of collagen II and glycosaminoglycan synthesis at high concentrations. Furthermore, our group showed for the first time that some chondrocytes which are present in OA cartilage are TH - positive. This indicates that the presence of sympathetic nerve fibers (in contrast to RA) and a high sympathetic activity might be beneficial in OA. However, no in vivo evidence exists at present confirming this hypothesis.

Taken together, all those reports discussed above are providing different and partly controversial data which do not allow a clear prognosis in which way sympathetic neurotransmitters contribute to chondrogenesis of progenitor cells and in mature chondrocytes to articular cartilage repair in both a non-inflammatory and an inflammatory microenvironment.

## 5. Sensory and Sympathetic Neurotransmitters and Their Receptors in Subchondral Bone

### 5.1. Sensory Neurotransmitters

Disturbed skeletal homeostasis has been observed in patients suffering from stroke, spinal cord injuries and other nerve injuries [80,81]. Degradative biomarkers from serum of these patients implied an uncoupling of the bone remodeling process in favor of bone resorption. The effect was mainly attributed to an unloading of the affected limbs but animal studies in rats using guanethidine-induced reduction in VIP and NPY levels showed induced osteoclastogenesis in the mandible without affecting periosteal bone formation [82]. Furthermore, surgical and chemical sympathectomy in Mongolian gerbils led to enhanced osteoclast resorptive activity in ear bones which are not normally subjected to loading [83]. Clearly, mechanisms not directly related to loading are responsible for skeletal changes following denervation. Numerous studies demonstrated the expression of receptors for a wide variety of neurotransmitters and neuropeptides on bone cells like osteoblasts and osteoclasts and tried to elucidate the influence on their cellular activities. To date, there are major limitations regarding our understanding of the influence of changes in innervation pattern of subchondral bone on the remodeling processes of bone observed in OA. So far, observations from studies of neuronal influence on bone cells can serve as references but gaining a clearer insight into actual pathological neuronal–skeletal interactions in OA will require considerable research efforts in the future.

#### 5.1.1. Substance P Effects on Osteoblasts

The extensive distribution of SP in joint tissues and also bone has been described earlier, including NK1-R, which is widely expressed in bone cells as osteoblasts, osteoclasts and osteocytes indicating a modulatory capacity of SP in bone remodeling. Goto et al. detected NK1-R expression in the cytoplasm and the plasma membrane of rat osteoclasts [84]. Compared to osteoclasts, in osteoblasts and osteocytes weaker immunoreactivity for the NK1-R was observed. However, missing additional studies evaluating the function of the receptor in these cell types makes it difficult to draw conclusions from the differential distribution of the NK1-R between osteoblasts and osteoclasts. In a very early study by Bjurholm et al., SP (and NPY) was not able to induce a cAMP response in rat osteosarcoma cell lines UMR-101-01 and ROS 17/2.8, the human osteosarcoma cell line Saos-2, a mouse calvarial pre-osteoblastic cell line, MC3T3-E1, and in primary mouse neonatal calvarial bone cells, indicating a minor role for SP in osteoblast function regulation [85]. However, work from Azuma et al. instead showed that SP enhanced the *Porphyromonas gingivalis* lipopolysaccharide-induced inhibition of bone nodule formation and alkaline phosphatase (ALP) activity in rat calvarial osteoblasts [86]. In contrast, various studies addressing SP effects on osteoblastic cells reversed these findings. Goto et al. reported an upregulation of the NK1-R in primary rat calvarial osteoblastic cells after 14 days of osteogenic differentiation, but not after 7 days, that promoted bone formation upon addition of SP to the culture medium [87]. In late-stage osteogenesis, addition of SP to the cell culture medium also stimulated osteocalcin, runt-related transcription factor (Runx) 2 and collagen I expression, but not in the early differentiation stage. From that observation the authors concluded an influence of SP on more mature osteoblast cells rather than on pre-osteoblastic cells, explaining some of the controversial findings indicated above as some of the cell lines investigated in the study by Bjurholm et al. represent rather early osteoblastic stages. Meanwhile, a number of investigations showed that SP promotes upregulation of the osteogenic transcription factor osterix during osteogenic differentiation of mesenchymal stem cells [88], activates the pro-osteoblastic Wnt-signaling pathway in MC3T3-E1 cells [89] and stimulates human osteoblastic cell activity by enhancing gap junction intercellular communication [90].

In a very recent study, Kodama et al. describe a bi-directional communication of osteoblastic MC3T3-E1 cells and dorsal root ganglion-derived sensory neurons [91]. The authors show that the efferent signal is transmitted via SP and glutamate and that osteoblast-like cells communicate to the afferent neural arm via adenosine triphosphate (ATP) exocytosis after perception of an inflammatory stimulus like bradykinin. From these observations, the authors hypothesize that osteoblasts and possibly other bone surface cells might serve as sensors for environmental stimuli and transmit this perception to the central nervous system via afferent nerves. These novel observations might broaden our knowledge and add even more complexity to the regulatory circuits involved in bone homeostasis, and have to be taken into consideration when evaluating studies targeting neuronal effects in the skeleton.

#### 5.1.2. Substance P Effects on Osteoclasts

While SP effects differ in early and mature osteoblasts, there is a consensus that SP stimulates osteoclastogenesis and favors bone resorption either directly [92,93] or indirectly by upregulation of the major osteoclast differentiation factor receptor activator of the nuclear factor (NF)-κB ligand (Rankl) in various cell types [94,95]. Contrary to the physiological situation, information on the effects of SP in bone in osteoarthritic conditions is sparse. Xiao et al. showed an increase in mean optical density for SP immunoreactivity (and also CGRP and VIP) in the cancellous bone of OA femoral heads compared to osteoporosis samples, which correlated positively with pain intensity analyzed by visual analog scale (VAS) but also with bone structural parameters analyzed by micro computer tomography (μCT) [96]. Concluding from this, SP might be implicated in OA pain but also seems to rather preserve bone structure in OA pathophysiology. Zhen and co-workers elegantly demonstrated that anterior cruciate ligament transection leads to spatiotemporal uncoupling of bone remodeling with an increase of osteoblast and osteoclast activity in an OA mouse model [97]. How local release of SP in bone tissue might be involved needs to be further elucidated, but acting as enhancer on both cell types, SP could contribute to these observations and SP targeted therapies could potentially target OA bone phenotypes too.

#### 5.1.3. CGRP Effects on Osteoblasts and Osteoclasts

Expression of the main CGRP receptor complex composed of CRLR and receptor activity modifying protein (RAMP) 1 (its co-receptor) has been demonstrated on several osteoblastic cell lines like MC3T3-E1 and MG63, rat primary calvarial osteoblasts and human primary osteoblast cultures [68,98,99,100]. Addition of CGRP to bone marrow stromal cells, subjected to osteogenic differentiation, enhanced proliferation and expression of osteoblastic genes like *Runx2*, *ALP*, *osteocalcin* and *col1a1* [101]. CGRP stimulation dose-dependently induced cAMP production and affected intracellular Ca^2+^-level in primary osteoblastic cells isolated from calvaria of newborn rabbits [102]. In addition, it induced the expression of osteoblastogenic activating transcription factor-4 (ATF-4) and osteoprotegerin (OPG) while decreasing expression of Rankl. Increasing the OPG/Rankl ratio and thus favoring osteoblast differentiation would shift the skeletal balance towards bone formation and emphasizes the anabolic character of CGRP. The indirect inhibitory effect of CGRP on bone resorption was additionally confirmed in the MC3T3-E1 pre-osteoblastic cell line where it downregulated Rankl and upregulated OPG, independently of mechanical stimulation [103]. Supporting the preserving effect of CGRP on bone matrix, different studies reported an inhibitory influence of CGRP on osteoclastogenesis. CGRP inhibited IL-1 induced osteoclastic bone resorption in a co-culture setup with osteoblasts on ivory slices either directly or indirectly [104]. In fetal rat osteoblasts, CGRP was able to inhibit the lipopolysaccharide (LPS) and IL-1-induced production of TNF and weakly induced IL-6 in these osteoblasts [105]. These inhibitory effects seemed to be not only mediated by classical cAMP-dependent pathways, but also the protein kinase C pathway. The indirect inhibitory effect of CGRP on osteoclastogenesis was demonstrated by regulating the Rankl/OPG ratio in osteoblast-like cells. Besides osteoblasts, osteoclasts and their precursors, bone marrow-derived macrophages (BMM), also express the CGRP receptor complex of CRLR and RAMP1 [106]. In addition, RAMP2 and 3 mRNA and protein expression was detected in osteoclasts, which points to regulatory control from other proteins of the calcitonin family apart from CGRP, i.e., amylin, adrenomedullin and intermedin. Wang et al. reported that CRLR immunostaining was more intense in BMMs and pre-osteoclasts compared to mature osteoclasts [101]. When they subjected BMMs to macrophage colony-stimulating factor (M-CSF)/Rankl induced osteoclast differentiation under CGRP stimulation, the cell cultures yielded reduced osteoclast numbers, erosion areas on osteologic discs were smaller and the mRNA expression of tartrate-resistent alkaline phosphatase (TRAP) and cathepsin K, two marker genes for mature osteoclasts, was reduced.

With regard to a complex disease like OA, every patient has some variation in subchondral bone and neuronal phenotype and, in many other aspects, variations in distribution of SP- and CGRP-positive nerves or synovial fluid/serum concentrations of these neuropeptides might correlate with individual subchondral bone radiographic status due to their partly opposite effects on bone cells.

### 5.2. Sympathetic Neurotransmitters

A similar complexity for neuronal regulation of bone cell function and differentiation is observed for neurotransmitters of the sympathetic nervous system like VIP, NPY and NE. In particular, NE potentially exerts a wide range of effector functions because of the multitude of receptors used by this neurotransmitter. Functional expression of the adrenoceptors α1B and β2 was detected by reverse transcription polymerase chain reaction (RT-PCR) and immunofluorescence staining in human osteoblast cells by Huang et al. [107]. Earlier studies were able to prove α- and β-AR expression mainly on gene expression level [98]. Protein levels of the α-receptors were low, consequently an influence of α-adrenergic regulation of bone cells remained questionable. An in vitro study using intertrochanteric trabecular bone samples from osteoporotic- and OA patients also detected the α2A-AR via immunostaining in cuboidal shaped osteoblasts and bone lining cells but not on osteocytes [108]. Pharmacological targeting of α-AR elucidated the role of these receptors in osteoblast-like cells. Usage of the α-AR agonists cirazoline and phenylephrine and the β-AR-blocker propranolol induced the proliferation of human osteoblasts whereas fenoterol, a β2-AR-agonist, inhibited osteoblast proliferation. Both agonists also dose-dependently enhanced the expression of Rankl and OPG, thus indicating an indirect regulatory influence on osteoclasts [107] (Figure 4). In the human osteoblast SaM-1 cell line, NE was able to induce cell proliferation via blockade of a potassium channel using a G_i/o_ signaling pathway. The effect was blocked by chloroethylclonidine, an α1B-AR antagonist [109]. The pro-osteoblastic effect of α-AR agonism was also observed by Tanaka and co-workers [110]. They reported that phenylephrine, a non-specific α1-AR agonist, induced the transcription factor CCAAT/enhancer-binding protein δ (CEBPD) in the pre-osteoblastic cell line MC3T3-E1 enhancing the proliferation of these cells. These studies indicate that noradrenergic signaling via α-ARs is primarily a positive regulator of osteoblast differentiation and an indirect inhibitor of osteoclasts.

Primary neonatal mouse calvarial osteoblasts from fluorescent ubiquitination-based cell cycle indicator (FUCCI) transgenic mice, expressing red nuclear fluorescence markers in osteoblasts in the G1 phase of the cell cycle and green fluorescence markers in the G2/M phase, were analyzed for the effects of the β-AR-agonist isoproterenol on osteoblast proliferation and migration. The isolated osteoblasts were capable of bone nodule formation in osteogenic medium, stating that the genetic engineering did not affect normal bone formation behavior. Isoproterenol suppressed migration velocity and distance and delayed cell cycle transition and thus proliferation, indicating that these effects add to disuse-induced bone loss [111]. Disuse-induced bone loss is a major side effect in age-related diseases leading to osteoporosis. The sympathetic tone has been discovered as a major origin of the disease-inducing signal [24]. When MC3T3-E1 cells were treated with the β-AR-agonist isoproterenol, adrenoceptor signaling significantly suppressed a bone morphogenetic protein (BMP)-2 induced increase in ALP expression in these cells [112]. Additionally, β-adrenergic stimulation led to enhanced Rankl expression in the osteocytic cell line MLO-Y4 which in turn induced osteoclastogenesis in osteocyte–RAW264.7 co-cultures [113]. Opposite effects of noradrenergic α- and β-AR signaling have been reported eliciting pro- and anti-inflammatory effects for RA and likewise changes in sympathetic innervation pattern in OA would provide an altered local neurotransmitter milieu. Thus, bone cell regulation could change and/or induce aberrant remodeling of subchondral bone.

Work from our group detected the protein expression of adrenoceptors α1D, α2B and β2 on mature osteoclasts as well as on rat BMM, the osteoclastic precursor population [62,114]. We have shown that α-adrenergic stimulation with 10^−8^ M NE increased osteoclast differentiation whereas β-adrenergic stimulation with 10^−6^ M instead decreased osteoclastogenesis (Figure 4). Furthermore, NE increased caspase 3/7-mediated apoptosis in the mixed cultures of BMM and osteoclasts. When rat BMMs were removed from a highly inflammatory environment, like in inflammatory arthritis, and subjected to M-CSF/Rankl-driven osteoclastogenesis, their reactivity to NE was altered with regard to osteoclast numbers, apoptosis and especially cathepsin K activity [62]. Although in OA inflammation is less prominent compared to RA, inflammatory mediators in joint adjacent bone compartments might influence neurotransmitter reactivity of local osteoclasts, thereby promoting alterations in bone metabolism. In contrast to our study and in support of earlier studies, Aitken et al. showed that β2-AR stimulation enhanced osteoclastogenesis indirectly as well as directly [115,116]. Work by Kondo et al. demonstrated that the osteoclastogenic effect of β2-AR stimulation was at least partly mediated by induction of reactive oxygen species [117]. These conflicting observations might be due to different stimulation regimen which addressed early and late stage differentiation of osteoclasts comparable to the aforementioned differential effects of SP on early and late stage osteoblastogenesis.

Information on changes of bone innervation during OA pathogenesis is rare. Studies using OA as comparative controls for respective analysis in RA patients where sympathetic nerve fibers get lost, show that presumably sympathetic innervation is less affected by OA-induced changes than sensory innervation. Inflammatory mediators are also evident in OA, although to a lesser extent than in RA. As we have shown in our work, inflammatory conditions might change the reactivity of osteoclast precursors thereby evoking an altered bone metabolism [62,114]. Thus, one might hypothesize that not necessarily innervation patterns change but the reactivity of cells by either modulating receptor expression or alteration of intracellular signal transduction.

Receptors for the neuropeptide VIP and its related moleculepituitary adenylate cyclase activating peptide (PACAP), mainly associated with anti-inflammatory reactions, were also detected on osteoclasts and osteoblasts of different species like human, mouse and rat as reviewed by [118]. In osteoblasts, VIP application elicited a cAMP response and led to induction of *ALP* gene expression and activity (Figure 4), stimulated IL-6 expression and increased the stimulatory effect of a number of cytokines on IL-6 production. In a co-culture system, VIP caused a delayed enhancement of osteoclast resorptive activity but in M-CSF/Rankl-induced osteoclastogenic cultures of BMM, VIP inhibited osteoclast formation. From that observation, the authors concluded that VIP regulates the expression of osteoclastogenic factors in osteoblasts [119]. A recent review by Juhasz et al. further highlighted the role of VIP in osteogenic signaling pathways [120]. VIP upregulates the transforming growth factor (TGF) β/BMP signaling pathway and the pathway’s effector molecules, SMADS, can in turn regulate VIP expression, leading to more complex reciprocal regulatory mechanisms. VIP is also implicated in direct activation of the ERK1/2 pathway in osteoblasts, thereby enhancing the Rankl/OPG ratio. From this activation of the ERK signaling pathway the authors deduced that VIP might also promote osteoblastogenesis like it was shown for activation of the fibroblast growth factor receptor 2. However, the authors provided no direct evidence for comparable VIP effects. Our group detected mRNA expression of VIP receptor 1 and 2 and the alternative VIP receptor PACAP receptor 1 in mixed cultures of osteoclasts and BMMs [62]. We observed that VIP had no profound effect on osteoclast numbers, but VIP-treated cultures showed a significantly reduced cathepsin K activity in the cell culture supernatant (Figure 4). VIP actions indicate an anabolic function in the skeletal system and protective effects of VIP application preventing destruction of bone and cartilage have been demonstrated in collagen-induced murine arthritis [121]. Therefore, VIP appears as a very attractive therapeutic option in OA.

The osteoblastic actions of NPY were recently very extensively reviewed by [122]. NPY actions on bone are not only derived from central neuronal signals but also from a highly complex peripheral network. Peripheral and thus bone resident NPY can be released from sources like sympathetic nerves, the adrenal medulla, pancreatic tissue or osteoblasts and osteocytes themselves, which produce NPY endogenously. The local production of NPY is involved in regulation of bone homeostasis by suppressing bone formation and modulating osteoblast progenitor commitment via the Y1 receptor. Most of the work in this review describes results from mouse experiments. In isolated human MSC under osteogenic conditions, NPY directly promoted osteogenesis by up-regulation of Runx2 and an increase in ALP-activity and Alizarin red staining (Figure 4). In contrast to mouse studies, the Y1 receptor mRNA was upregulated in the course of osteogenic differentiation of human MSC [123]. There might be a discrepancy in NPY receptor response in various species that needs consideration when evaluating new treatment targets in whole joint diseases like OA. Studies on NPY and osteoclasts are rare and so far, only one study describes that BMM cultures from mice lacking expression of the NPY receptor y6R showed an increase in M-CSF/Rankl induced osteoclastogenesis [124], indicating that NPY actions are preservative in the bone environment.

## 6. Perspectives

Sensory and sympathetic nerve fibers and their neurotransmitters are important neuronal effectors regulating cartilage and bone physiology and playing decisive roles in musculoskeletal pathophysiologies. Notably, many resident cells of the osteoarticular system have receptors for sympathetic and sensory neurotransmitters, thus, they can respond to these stimuli. Embryonic limb growth and post-natal long bone growth is modulated via sympathetic and sensory neurotransmitters by targeting chondrocytes in the growth plate. It becomes more and more evident that neuronal signaling critically influences tissue regeneration, i.e after bone and meniscal traumata and tendon/ligament ruptures. However, it is not well understood and discussed controversially in literature how changes in sensory and sympathetic nerve fiber profile and their respective neurotransmitters contribute to abnormal subchondral bone remodeling, cartilage degradation and osteophyte formation during the pathogenesis of OA.

Taken together, it becomes more and more evident that sensory and sympathetic nerve fibers and their neurotransmitters critically influence cartilage, subchondral bone, and other joint tissue function and homeostasis. Without doubt, the peripheral nervous system is crucially involved in the pathogenesis of musculoskeletal disorders such as OA and others and cannot be ignored in the analysis of underlying molecular mechanisms.
